# miR-218: A Stress-Responsive Epigenetic Modifier

**DOI:** 10.3390/ncrna8040055

**Published:** 2022-07-21

**Authors:** Grant Schell, Bhaskar Roy, Kevin Prall, Yogesh Dwivedi

**Affiliations:** Department of Psychiatry and Behavioral Neurobiology, Heersink School of Medicine, University of Alabama at Birmingham, Birmingham, AL 35294, USA; gvs1012@uab.edu (G.S.); bhaskarroy@uabmc.edu (B.R.); kevinprall@uabmc.edu (K.P.)

**Keywords:** microRNA, miR-218, stress, neuropsychiatry, depression, human brain, animal models

## Abstract

Understanding the epigenetic role of microRNAs (miRNAs) has been a critical development in the field of neuropsychiatry and in understanding their underlying pathophysiology. Abnormalities in miRNA expression are often seen as key to the pathogenesis of many stress-associated mental disorders, including major depressive disorder (MDD). Recent advances in omics biology have further contributed to this understanding and expanded the role of miRNAs in networking a diverse array of molecular pathways, which are essentially related to the stress adaptivity of a healthy brain. Studies have highlighted the role of many such miRNAs in causing maladaptive changes in the brain’s stress axis. One such miRNA is miR-218, which is debated as a critical candidate for increased stress susceptibility. miR-218 is expressed throughout the brain, notably in the hippocampus and prefrontal cortex (PFC). It is expressed at various levels through life stages, as seen by adolescent and adult animal models. Until now, a minimal number of studies have been conducted on human subjects to understand its role in stress-related abnormalities in brain circuits. However, several studies, including animal and cell-culture models, have been used to understand the impact of miR-218 on stress response and hypothalamic-pituitary-adrenal (HPA) axis function. So far, expression changes in this miRNA have been found to regulate signaling pathways such as glucocorticoid signaling, serotonergic signaling, and glutamatergic signaling. Recently, the developmental role of miR-218 has generated interest, given its increasing expression from adolescence to adulthood and targeting the Netrin-1/DCC signaling pathway. Since miR-218 expression affects neuronal development and plasticity, it is expected that a change in miR-218 expression levels over the course of development may negatively impact the process and make individuals stress-susceptible in adulthood. In this review, we describe the role of miR-218 in stress-induced neuropsychiatric conditions with an emphasis on stress-related disorders.

## 1. Introduction

Neuropsychiatric disorders such as major depressive disorder (MDD), bipolar disorder (BD), schizophrenia (SZ), and anxiety are specific, clinically recognized conditions in which an individual’s thoughts, perceptions, emotions, and cognition are affected [1]. These complex changes are primarily due to underlying brain pathologies acquired over a period of time [2]. Environmental stress is widely recognized as one of the main risk factors for neuropsychiatric conditions, where changes in cellular interface are often associated with abnormal signal processing in neuronal and nonneuronal circuits [3,4]. Abnormality in signal-processing pathways is usually due to overwhelming changes in large-scale gene regulatory functions [5]. Since epigenetic changes involve a wide range of molecular interplays, both at the intracellular and extracellular levels, a number of cellular modifiers have been traced at the core of this molecular process [6]. Of these, noncoding RNAs (ncRNA) are gaining attention for their role in various disease processes. ncRNAs are defined as RNAs that are not translated into proteins [7]. Protein-coding genes occupy only a small proportion (<3%) of the entire genome; however, the remaining non-protein-coding genes are not a simple transcriptional noise, as ~80% of them are transcriptionally active with various regulatory roles [8]. A majority of them are now considered ncRNA genes [7]. Based on the size of the nucleotides, ncRNAs are divided into small ncRNAs (sncRNA) and long ncRNAs (lncRNAs). ncRNAs containing <200 nucleotides (nt) belong to sncRNA families. On the other hand, those having >200 nt belong to the lncRNA family. Small ncRNAs include miRNAs, small interfering RNAs (siRNAs), piwi-interacting RNAs (piRNAs), small nucleolar RNAs (snoRNAs), and small nuclear RNAs (snRNAs) [9,10,11]. ncRNAs function via interactions with RNA, DNA, and protein. They regulate the transcription of messenger RNAs (mRNAs) and participate in alternative splicing and epigenetic modifications such as chromatin and RNA editing [12,13]. These regulatory functions can target either neighboring transcripts (cis) or loci distant from their own transcription (trans). Collectively, ncRNAs are a unique layer of gene regulatory molecules that function as key intermediate regulators in conveying the message from genotype to phenotype states [11,14]. Of various ncRNAs, miRNAs are the most curated, characterized, and studied in both humans and animals [15]. With their enormous ability to push and pull the transcriptional and posttranscriptional output of gene expression, miRNAs can determine an altered epigenetic landscape [16,17]. In fact, their inherent ability to bring about reversible changes in gene expression is collinear with improper functioning of the central nervous system (CNS) when challenged with aversive environmental stimuli [18]. The environmental insults are common to many neuropsychiatric conditions, including MDD, BD, and SZ [19]. They are considered as one of the primary triggers in repatterning the neural information-processing pathways [20].

Until recently, the role of miRNAs has been less appreciated in mental disorders [21]. Skepticism of using ncRNAs as clinical biomarkers also undermined their high predictive value in diagnosing neuropsychiatric conditions [22]. However, research in the last decade has drawn substantial attention to accepting the critical role of these sncRNAs in repatterning the epigenetic circuitry and their role in psychiatric illnesses [23]. Compelling evidence based on preclinical and clinical studies has shown that circulating miRNAs in peripheral blood have the potential to proxy brain-associated changes [24]. Emerging evidence has also supported the pathogenic role of miRNAs in the development and progression of various neuropsychiatric conditions [25].

Many miRNAs are considered stress-associated with their direct or indirect influence on the functionality of the central stress axis in the brain [26,27]. One such stress-responsive miRNA is miR-218 which has recently emerged with functional roles in modulating the signaling pathways and related gene expression in animal stress models [28]. Increasing focus on miR-218 has been associated with various brain functions, including synaptic plasticity, cell survival, and behavior [29,30]. Our group and other investigators have shown that this miRNA is involved in stress and susceptibility to developing depression and can be used as a biomarker for diagnosing stress-related disorders [31,32,33]. In this review, we have provided an overview of the importance of studying miRNAs in brain functions and how they are epigenetically connected with various neuropsychiatric abnormalities, especially depression and other stress-related mental disorders. Later in the review, based on our current understanding, we have specifically highlighted the role of miR-218 in stress pathology and its association with stress-related mental disorders, including MDD.

## 2. miRNA Biogenesis following Canonical Pathways

MicroRNA is one of the candidates from the sncRNA family with a precise epigenetic role in modulating the coding potential of the transcribed mRNA pool based on characteristic sequence complementarity [34]. Since their first report as epigenetic modifiers, miRNAs have evolved from being a modulator of a single protein-coding gene to acting as a potential regulatory hub to control a wide array of complex gene networks either through direct association or by indirect intermediates [35]. Despite the restricted size (~22 nucleotides) and limited potential to go through the exon-splicing (involving the removal or “splicing out” of specific sequences referred to as intervening sequences or introns, which allows the joining of two spanning exons by forming a lariat-like structure) procedure for generating more structural variations [36], this small-form factor exhibits a functional diversity in targeting a diverse range of RNA molecules spanning from protein-coding (mRNA) to long noncoding RNAs (lncRNAs) [37]. Mammalian miRNA biogenesis follows a programmed pathway to produce the mature effector molecule mediated by two ribonuclease (RNase) III enzymes, Drosha and Dicer [15]. First, miRNAs are converted to precursor transcripts (pre-miRNA) from primary miRNAs by Drosha, a nuclear RNase III enzyme, which is then exported to cytosol and processed by the RNase III enzyme Dicer [38,39]. Drosha is part of a multiprotein complex, the microprocessor, which mediates the nuclear processing of the primary miRNAs into stem-loop precursors of approximately 60 to 70 nucleotides (pre-miRNA). Drosha cleaves the RNA duplex with a staggered cut so that the base of the pre-miRNA stem-loop has a 5′ phosphate and ~2 nt 3′ overhang [40,41]. As the nuclear-cut Drosha defines one end of nascent miRNA, the other end is defined by Dicer in the cytoplasm. In doing so, it recognizes the affinity of pre-miRNAs toward a 5′ phosphate and 3′ overhang at the base of the miRNA stem-loop [42,43]. In humans, ~3000 mature miRNAs have been annotated; about 30% of them reside in the intronic regions of protein-coding genes [44]. However, the miRNA loci are often mapped with distinct transcriptional units in the mammalian genome, mainly using RNA polymerase II enzyme for transcription [45].

## 3. miRNAs as Epigenetic Regulators of Brain Functions and Dysfunctions

**Figure 1:**
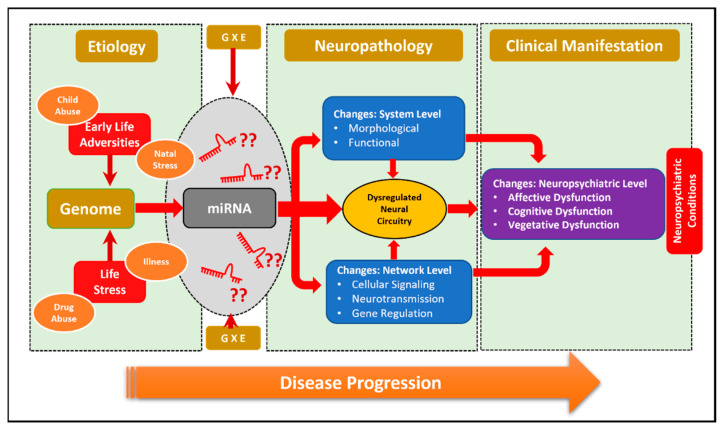
miRNAs as stress-induced epigenetic regulators of neuropsychiatric disorders. Gene and environmental (GXE) crosstalk shape the miRNA-based epigenetic landscape of the brain in neuropsychiatric disorders, largely triggered by various stress-inducing factors.

Biologically, stress-associated neuropsychiatric conditions demonstrate maladaptive stress-responsive pathways due to homeostatic imbalances in the brain [46]. It is known that many of the changes associated with homeostatic imbalances can be partly dictated by a wide range of miRNA molecules targeting an array of gene regulatory phenomena, including pre- and post-transcriptional modifications [47]. Many psychopathologies associated with neuropsychiatric conditions are primarily contributed by gene-expression changes in the synaptic compartment and neuronal soma [48]. Parallel changes in miRNA expression have also been identified and have drawn significant attention due to their localized distribution in synaptic and dendritic areas [49]. It is interesting to examine if their discrete compartmentalization has a vital role in activity-dependent gene regulation linked with synaptic plasticity (refers to the activity-dependent modification of the strength or efficacy of synaptic transmission at preexisting synapses) [50]. Large-scale miRNA sequencing data from our lab has recently clarified that the differential distribution of miRNAs between neuronal soma and the synaptic chamber is closely associated with neuronal functions in the MDD brain with an explicit role in synaptic plasticity [49]. Another noticeable feature of the brain-enriched miRNAs is the genetic redundancy in their coding unit. For example, the transcriptional unit of the same miRNAs was found to be present on different arms of the same chromosome or localized on different chromosomes [51]. It is remarkable that for the brain to function under complex neuronal inputs, miRNAs act as a tool for the regulatory mechanisms that control large-scale gene expression changes [52]. Understanding miRNA activity has undoubtedly helped to examine the role of miRNAs in regulating neurogenesis, synapse development, axon guidance, neuronal plasticity, and abnormalities occurring due to pathological changes in the neuropsychiatric brain (Figure 1) [53]. In addition to their role in the CNS, miRNAs are found to reflect changes in the peripheral circulation. Empirical data show that miRNAs can be used to diagnose disease conditions and in the treatment response [54].

## 4. Role of miRNAs in Neuropsychiatric Disorders

A wealth of data show that miRNAs play an essential role in neuropsychiatric disorders such as MDD, SZ, and BD [21,55,56]. Several studies have linked miRNA changes to MDD. In a paper by Dwivedi [57], it was noted that 29 miRNAs were downregulated in the dorsolateral PFC (dlPFC) of MDD subjects. Many of the targets for these downregulated miRNAs were signaling proteins, nuclear proteins, transmembrane proteins, and transcription factors [16]. Dysregulation of miRNAs was also observed in rats that underwent early-life stress and displayed a depressive phenotype in adulthood [58]. In Brodmann Areas 9, 10, and 44, miRNA dysregulation has been identified in depressed populations [59]. In BA9, both miRNA-30a-5p and miRNA-30e-5p were upregulated alongside a corresponding downregulation of the target gene ZDHHC21 [59]. This gene plays a critical role in palmitoylation of serotonin autoreceptor 1A, a deficit observed in the tissue of depressed suicide samples and mice displaying a depressive phenotype [59]. In BA44 from depressed individuals, upregulation of numerous miRNAs was noted. These include miR-34c-5p, miR-139-5p, miR-195-5p, and miR-320c. In mouse models of depression, miR-139-5p was also upregulated. Interestingly, the depressive phenotype was alleviated when the mice were administered a miR-139-5p antagomir intranasally [59]. In the ventrolateral PFC (vlPFC) of depressed individuals, miR-1202 was the most differentially regulated miRNA, which regulates the expression of a gene-encoding metabotropic glutamate receptor-4 (GRM4) [60]. Interestingly, the GRM4 3′ UTR variant (rs2229901) is associated with MDD risk [61]. Both human and animal studies suggest that miR-124 expression is regulated by stress and is critical in developing depression [62]. Another study analyzed 29 select miRNAs by qPCR in the anterior cingulate cortex (ACC) region of MDD, BD, and control subjects. They found that miR-132, miR-133a, and miR-212 were differentially regulated in BD, miR-184 in MDD, and miR-34a in both MDD and BD [63]. Several studies have shown miR-34a to be upregulated in BD [64]. This miRNA is believed to play a role in G-protein signaling, apoptosis, and calcium regulation. A different study of the human postmortem PFC showed significant dysregulation of miRNA in BD, with 19% of 234 miRNA showing differential expression [65]. A study by Camkurt et al. [66] found significant upregulation of four miRNAs in bipolar patients, miR-29a-3p, miR-106b-5p, miR-17, and miR-125a-3p. Additionally, when only bipolar manic patients were compared to controls, seven miRNAs were significantly upregulated, while two miRNAs were significantly upregulated in euthymic patients compared to control. That said, all miRNAs in euthymic patients were observed to be at least slightly upregulated. In both euthymic and manic bipolar patients, miR-107 was upregulated. This miRNA is predicted to target two glutamatergic genes, GRIN2A and SLC1A4, thus causing a deficit of glutamatergic transmission [66].

A meta-analysis conducted by Thomas and Zakharenko found that four miRNAs (miR-941, miR-199a-3p, miR-92a-3p, and miR-31-5p) were downregulated, and one miRNA (miR-103a-3p) was upregulated in subjects at high clinical risk of SZ patients that progressed to psychosis [67]. The prediction by TargetScan (Human) shows that these miRNAs likely target genes associated with neuronal function and plasticity. Attesting to a role in neuronal function, miR-103a-3p was used as part of a classifier function to predict cortical thinning. In patients at high clinical risk who proceeded to psychosis, higher-than-average rates of cortical thinning were found [67]. In the same study, normal levels of miR-223-3p were determined in the dlPFC of postmortem brain samples from SZ patients [67]. MiR-137 has also been identified as playing an important role in SZ. It targets several genes associated with SZ and originates from the genome region that is the second most statistically significantly altered in SZ subjects [68]. Another miRNA shown to have altered levels was miR-181b [69,70]. MiR-181b was upregulated with an associated downregulation of target genes in postmortem human superior temporal gyrus (STG) samples with SZ [69]. Several of the target genes are believed to be associated with the development of SZ [69,70]. Hauberg et al. [71] found that genes with more target sites for miRNA were more strongly associated with SZ. A study of 304 postsynaptic and 242 presynaptic proteins predicted that 91% were miRNA targets [70]. Given that cognitive abilities are dysfunctional in SZ, this prediction provides evidence of the role of miRNAs in SZ psychopathology.

Identifying stress-associated changes in miRNAs have been a key area of epigenetic research in the field of neuropsychiatric disorders [27]. Glucocorticoids play essential roles in stress system reactivity [72]. In the stress-axis system, in response to stress, corticotrophin releasing hormone (CRH) is released from the hypothalamus, where it binds to its receptors. This is followed by the release of adrenocorticotropic hormones (ACTH) from the pituitary. ACTH stimulates glucocorticoid synthesis in the adrenal cortex and releases them into the bloodstream. Glucocorticoids function after binding to two nuclear receptors, a-glucocorticoid receptor (GR) and a mineralocorticoid receptor (MR). In the brain, glucocorticoids participate in a negative feedback loop by binding to GR and MR in the hypothalamus [73]. Glucocorticoids are involved in various functional aspects of the central nervous system, such as behavior, emotion, and learning. Proper functioning of the HPA axis is key to stress responsiveness [74]. A recent study showed a transcriptome-wide change in miRNA expression in the PFC of rats treated with corticosterone [32]. Chronic corticosterone (CORT) administration caused a significant change in 26 miRNAs. Of them, 19 were upregulated (let-7i, miR-19b, miR-29c, miR-101a, miR-124, miR-137, miR-153, miR-181a, miR-181c, miR-203, miR-218, miR-324-5p, miR-365, miR-409-5p, miR-582-5p, miR-155, miR-29a, miR-30e, miR-721, miR-699) and 7 were downregulated (miR-146a, miR-200c, miR-351, miR-155, miR-678, miR-764-5p, miR-135a*). Our group showed that a subgroup of rats that displayed hopelessness had a blunted change in frontal cortical miRNAs compared to resilient rats [75], thus suggesting that aberrant miRNA expression can lead to deficits in the coping response to stress [56]. The stress-sensitive F344 rats, which show exaggerated release of CORT in response to a stressor, increased expression of hypothalamic miR-18a, which binds to 3′UTR of GR and reduces its expression [76]. This resulted in increased CORT release because of reduced feedback inhibition. In addition, exposure of neurons to excessive CORT resulted in a decrease in the BDNF-dependent neuronal function via suppression of miR-132 [77]. A recent report that miR-124-3p regulates the glutamatergic receptor system in stress-induced depression added another dimension to epigenetic regulation of genes that play a role in synaptic plasticity [62]. Another study of chronic unpredictable stress (CUS) showed a change in let-7a expression possibly mediated through repression of HTR4 gene expression in the hippocampus [78]. Studies have also found that miR-124 and miR-18a mediate downregulation of GR translation and play a role in susceptibility to stress [79]. In another study, rats undergoing chronic and acute immobilization stress showed changes in miR-134, miR-17-5p, and miR-124 expression in the cornu Ammonis 1 (CA1) region of the hippocampus and the central nucleus of the amygdala. These miRNAs are involved in regulating dendritic spine morphology [80,81,82]. A change in miR-34c in the central amygdala of stress-induced mice increased anxiety-like behavior [83].

Despite many studies in both human postmortem brain and animal models, the role of specific miRNAs in a particular neuropsychiatric disorder is still unclear. To a certain extent, it is understandable given that miRNAs target multiple genes simultaneously, some of which could be involved in one disorder but not in others [21]. On the other hand, overlapping miRNAs may appear in multiple psychiatric disorders. Given that MDD, BD, and SZ have distinct clinical and behavioral features, it is quite possible that miRNAs may target specific behavior by targeting select genes or pathways [84]. Nevertheless, it is clear from past studies that miRNAs play a significant role in various aspects of brain functioning, and their dysregulation may be part of the pathophysiology of psychiatric illnesses [55]. For example, we have shown that specific miRNAs in PFC are associated with resilience or susceptibility to stress, and any perturbations in these miRNAs may lead to stress-induced depression [75]. Another example is that viral vector-mediated overexpression of miR-124 in murine hippocampal neurons conferred behavioral resilience to chronic mild unpredictable stress (CMUS) [85] In contrast, the infusion of the anti-miR-124 enhanced vulnerability to stress. This study supports the idea that modulation of miR-124 may contribute to stress resilience (in case of overexpression) or vulnerability (in case of downregulation) [85]. This may be due to spatiotemporal changes in targeting downstream pathways or the result of other underlying epigenetic constructs that help develop coping strategies which render them more resilient [86]. A better understanding of how miRNAs are spatially and temporally expressed throughout development will also aid in determining which miRNAs are the most important for psychiatric illnesses [25]. In addition, more studies are needed to examine the cell-type specificity of miRNA changes, given that miRNA expression is brain-region- and cell-type-specific [87]. Sex-dependent changes are also essential to investigate. In a recent study, we reported that sex plays a critical role in the hypothalamic miRNA response to both early-life and acute stress, with males expressing greater changes following postnatal stress [88]. The studies of miRNAs in neuropsychiatric diseases are exciting not only from the viewpoint of understanding their molecular mechanisms, but also as biomarkers for diagnosis.

Multiple studies have explored the role of miR-218 in regulating stress responsiveness [28,89]. Since miRNAs form connections with other miRNAs to achieve regulatory functions [90], in this review, we have presented evidence that miR-218 can be mapped in the stress-responsive regulatory network in the brain with a possible relationship with additional stress-susceptible miRNAs.

## 5. Emerging Role of miR-218 in Disease Pathogenesis

**Figure 2:**
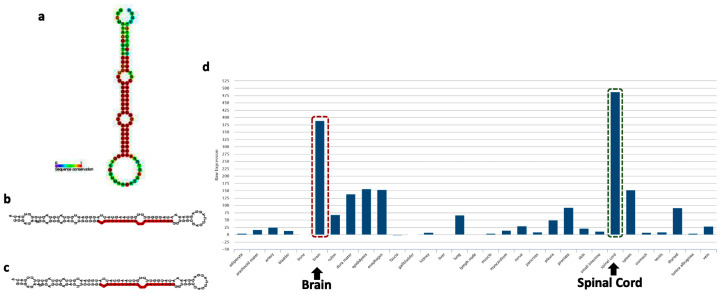
MiR-218 characteristics. Stem-loop structure of precursor miR-218 (**a**). The color scale shows the sequence conservation pattern within the stem-loop structure of the precursor miR-218. The independent stem-loop structure of miR-218-1 (**b**) and miR-218-2 (**c**). The expression atlas of mature miR-218-5p in different human tissue types. The highlighted bar shows the highest expression level observed in brain tissue besides the spinal cord (**d**).

MiR-218 has recently emerged as a key player in stress predisposition, as well as in its ability to deconstruct the information-processing pathways in the MDD brain by affecting diverse arrays of gene regulatory networks [28]. The miR-218 is miRTronic in nature. It means that miR-218 has a transcriptional origin from an intronic part of the coding gene. The characteristic stem-loop structure of precursor miR-218 has been shown on Figure 2a. The transcriptional origin of miR-218 has been mapped on two separate loci, miR-218-1 and miR-218-2 and found to be located at the introns of SLIT2 and SLIT3 genes, respectively [33]. More specifically, miR-218-1 is located within intron 15 of the SLIT2 gene, and mir-218-2 is present within intron 14 of the SLIT3 gene. The mature sequences for miR-218-1 and miR-218-2, termed miR-218, are identical [91]. The differences in sequence between the two isoforms of miR-218 are located at the 3′ end of the individual stem-loop structures (Figure 2b,c) [92]. In addition to its unique genomic organization, miR-218 also carries a signature expression mark in the central nervous system. According to the human expression atlas database [93], miR-218 is most abundantly expressed in the brain besides its expression in the spinal cord (Figure 2d). It is interesting to note that the gene organization and protein structures of SLIT2 and SLIT3 are very similar, as well as the location of the miR-218-1 and miR-218-2 stem loops. As reported in the miRBase database, the most abundantly expressed mature form of miR-218 is miR-218-5p (based on the normalized read counts from the next-generation sequencing data) [94]. In larger vertebrates, the mature miR-218-5p is processed from the miR-218-2 transcript, housed within the intronic region of the SLIT3 coding gene. It has earlier been shown that the other member of the SLIT gene family, i.e., SLIT2, harbors intronic miR-218-1, which encodes the other isoform of the miR-218 family (miR-218-1-3p). It is interesting to highlight that both isoforms are rooted in the Slit-Robo signaling axis with a functional role in depression-/anxiety-like behaviors as previously reported in adult mice [33]. The secreted Slit ligands and their Robo receptors constitute a signaling pathway that controls the directed migration of neurons and vascular endothelial cells during embryonic development. Still, the mechanisms of their regulation are incompletely understood [95]. A previous finding using both in-vivo and in-vitro models linked the SLIT gene expression to the posttranscriptional regulation of Robo receptors and heparan sulfate biosynthetic enzymes. The authors demonstrated that miR-218 directly represses the expression of ROBO1, ROBO2, and glucuronyl C5-epimerase (GLCE) and that an intact miR-218-Slit-Robo regulatory network exists [96]. In the brain, miR-218 is highly expressed in astrocytes and impacts gliomagenesis [97]. In addition, miR-218 has an impact on the development of motor neurons (MN) in the spinal cord and dopaminergic neurons in the midbrain. For instance, inhibition of miR-218 in the developing spinal cord can repress MN generation through the downstream Isl1-Lhx3 pathway, and mice with a complete loss of both miR-218-1 and miR-218-2 died neonatally and showed a significant loss of MN [98,99].

The development of midbrain dopaminergic (DA) neurons is a complex process that requires precise spatial and temporal expression of numerous genes, including the key transcription factor EBF3 [100]. Earlier data show that EBF3 is a candidate target for miR-218 during DA neuronal development. Since then, it has been established that the regulation of EBF3 expression by miR-218 controls the terminal differentiation of DA neurons [100]. Some of the other functions that require epigenetic regulation by miR-218 expand to neuritic projections and homeostatic regulation of synaptic plasticity. In this case, the regulation primarily happens through AMPA (α-amino-3-hydroxy-5-methyl-4-isoxazolepropionic acid)-mediated excitatory transmission. Studies have shown that miR-218 abundance is regulated during hippocampal development and by chronic silencing or activation of neuronal networks [29,30,101]. Overexpression and knockdown of miR-218 demonstrated that miR-218 targets the mRNA encoding the GluA2 subunit of AMPA receptors and modulates its expression. At the functional level, miR-218 overexpression increases glutamatergic synaptic transmission at both single neuron and network levels [29]. Besides controlling the functions in CNS, miR-218 was also explored as a new virus-induced miRNA that dampens the expression of RIG-I in mouse and human macrophages, leading to the impaired production of type I IFNs. Results have shown that interfering miR-218 expression rescued RIG-I-mediated antiviral signaling and thus protected macrophages from viral infection [102]. It is also interesting that miR-218 participates in cardiac stem cell differentiation. MiR-218 modulates the Wnt (Wingless/integrated) signaling in mouse cardiac stem cells by promoting proliferation and inhibiting differentiation through a positive feedback loop [103]. Finally, in a recent investigation, an additional role of miR-218 has been highlighted. In adolescent mice, an abnormality in miR-218 was seen as an early predictor of lifetime stress vulnerability in male mice [89].

## 6. Role of miRNA-218 in Stress Susceptibility

**Figure 3:**
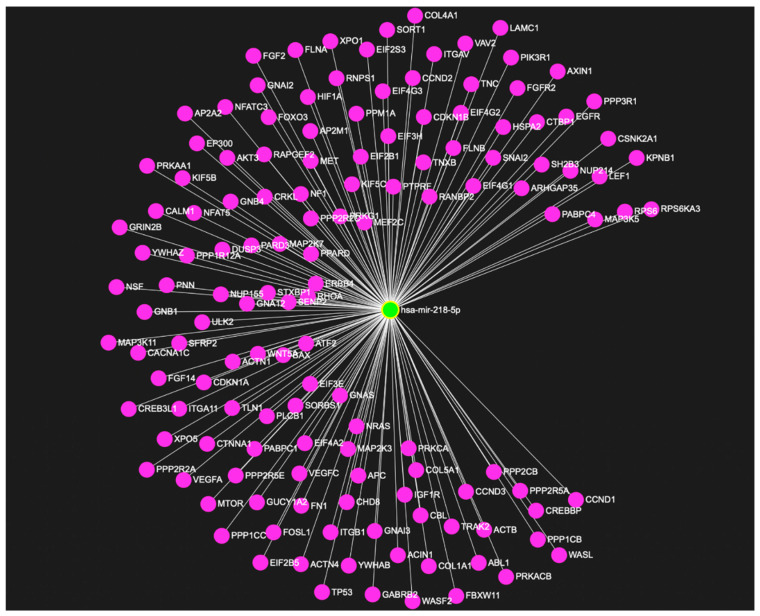
MiR-218-5p as a center of a diverse target gene-regulatory network enriched in the brain. The figure shows the regulation of various genes targeted by miR-218-5p.

**Figure 4:**
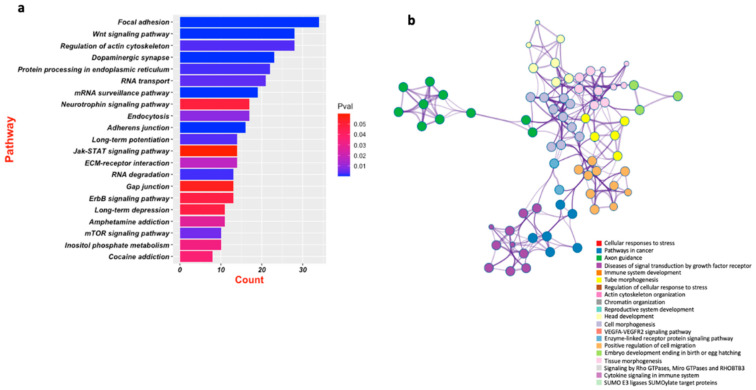
Brain-centric functional pathways based on an algorithmic determination of miR-218-5p target genes. Key pathways (bar plot) affected the central nervous system based on the brain-enriched predicted targets of miR-218-5p (**a**). The target pathways based on the predicted target of miR-218-5p are used to create a network map. The significantly enriched pathways from the network map, which are enriched in brain functions, are shown with color legends (**b**).

Multiple studies have explored the role of miR-218 in regulating stress response pertaining to depression [28,33,89]. A very recent study by our lab has made a significant contribution to understanding the role of this miRNA in tethering hundreds of key genes (Figure 3) implicated in central nervous system functions, notably synapse organization, neuron-projection morphogenesis, and axonogenesis (Figure 4a,b) [33]. The study highlighted the role that miR-218-5p plays in chronic stress and how it can contribute to acquiring maladaptive changes in the MDD brain through epigenetic dysfunctions. Dwivedi et al. found miR-218 to be highly upregulated in the PFC of CORT-treated rats, a rat model emulating chronic stress associated with depression [32]. Male Sprague Dawley rats received corticosterone injections or vehicle (control) for 21 consecutive days and underwent behavioral tests to assess the depressive phenotypes. CORT-treated rats showed significantly higher immobility time and significantly reduced sucrose consumption. MiRNA expression was measured using a Taqman low-density array and validated by quantitative PCR (qPCR) system. Predicted targets for statistically significant miRNAs were analyzed using Ingenuity Pathway Analysis (IPA) Software and validated via qPCR. The two most significantly dysregulated miRNA, miR-124 and miR-218, were both upregulated in the PFC of CORT-injected rats versus controls. MiR-218 was found to target the genes CREB1, MECP2, GRIA2, GRIA4, SP1, PIK3C2A, NFATC1, and GSK3β [32]. These genes have earlier been associated with stress and depression pathophysiology [104]. A follow-up study published in 2022 further explored how CORT-treatment mechanistically affected miR-218-5p expression and how miR-218 could trigger molecular changes in its downstream regulatory pathways [33]. A GR-targeted chromatin immunoprecipitation (ChIP) assay revealed high GR occupancy on the promoter region of the SLIT3 gene hosting miR-218-2 in its third intron. RNA-sequencing data based on RNA induced silencing complex immunoprecipitation (RISC) with AGO2 in SH-SY5Y cells detected several target genes such as APOL4, DTWD1, BNIP1, METTL22, SNAPC1, and HDAC6. Gene ontology (GO) analysis showed that most intergenic sites are part of these key genes implicated in CNS functions. These were: synapse organization, neuron-projection morphogenesis, and axonogenesis. These results suggest that the upregulation of miR-218-5p in CORT-treated rats possibly resulted from GR binding in the promoter region of the SLIT3 gene. Interestingly, miR-218 regulated HDAC6 potentially regulates CNS-related genes by chromatin modification. These studies demonstrate that miR-218 participates in the CNS stress response [32,33].

A series of studies also explored the participation of miR-218 in stress susceptibility through its regulation of the NETRIN-1 guidance cue receptor DCC (deleted in colorectal cancer) [29,31,105,106]. Here, the role of miR-218 has been ascribed as a molecular switch in priming the Netrin-1 signaling pathway. According to data, miR-218-mediated changes in Netrin-1/DCC signaling may lead to stress susceptibility and neuropsychiatric abnormality. The Netrin-1/DCC signaling pathway plays a role in the development and function of the PFC [31]. Torres-Berrio et al. analyzed the expression of miR-218 and DCC in postmortem BA44 of a cohort of depressed subjects who died by suicide and healthy, sudden-death control subjects [106]. They found that DCC expression was increased while miR-218 expression was decreased in the PFC of depressed individuals compared to controls [106].

Additionally, miR-218 and DCC expression were analyzed in the medial PFC (mPFC) of adult male C57BL/6 mice subjected to a chronic social-defeat stress paradigm (CSDS). Again, DCC expression was increased, and miR-218 expression decreased in stress-susceptible mice compared to control and resilient mice. Transfection of IMR-32 cells (human neuroblastoma cell line) with a miR-218 mimic demonstrated the ability of miR-218 to regulate DCC [106]. Torres-Berrio et al. followed up with another experiment using the CSDS model [28]. MiR-218 expression in the mouse PFC was modified by stereotaxic injection of either an antagomiR (anti-miR-218) that represses the miRNA’s activity or an adeno-associated viral construct that induces overexpression. Mice with increased miR-218 activity were more stress-resilient, while those with decreased miR-218 activity were more prone to develop depression-like behaviors. Additionally, levels of circulating miR-218 were positively correlated with miR-218 expression in the mPFC [28].

Torres-Berrio et al. took their research one step further by testing whether the expression of DCC and its regulator, miR-218, change throughout development and maturation [89]. The same CSDS model was employed with C57BL/6 male mice, but samples were collected in early adolescence (postnatal day, PND 21) and mid-adolescence (PND 35) in addition to adulthood (PND 75). The authors reported a trend of increasing miR-218 expression and decreasing DCC in the mPFC from early adolescence to adulthood, which illustrates that miR-218 plays a role as a regulator of DCC over the course of development. As with their previous finding, stress-resilient adult mice showed higher levels of miR-218 relative to their stress-susceptible counterparts. However, susceptible mice were found to have higher “adult-like levels” of expression of miR-218 in adolescence that were reduced after CSDS in adulthood. Stereotaxic injection of anti-miR-218 at approximately PND 40 allowed for the artificial reduction of miR-218 expression during development, resulting in a higher proportion of stress-resilient individuals in adulthood [89].

While testing the antidepressant effects of N3-polyunsaturated fatty acids (n3-PUFA), Kim et al. reported differential expression of miR-218 in the hippocampus related to HPA axis activity [107]. Wistar rats were bred and supplied with either a control diet or a diet supplemented with 1% n-3 PUFA. The supplemented diet was given either during the pre-weaning period (from the dam’s gestation day to postnatal day 20), the post-weaning period (PND 20–94), or for their complete lifetime (from gestation to PND 94). The animals were additionally separated into groups of non-stressed individuals who underwent chronic mild stress (CMS) or a combination of maternal separation and chronic mild stress. For maternal separation, pups were separated for 3 h per day from PND 2–14. CMS took place from PND 77–91, involving a rotation of stressors such as food and water deprivation, soiled bedding, and interrupted light–dark cycles. The analysis of various groups found that the n3-PUFA-supplemented diet reduced depressive behavior and reduced plasma levels of ACTH and CORT. Hippocampal expressions of multiple genes were altered compared with the control diet, such as increased CREB, GR, and BDNF and decreased TNF-α or IL-6. MiRNA analysis found that these group differences were also accompanied by the differential hippocampal expression of miR-218; stressors elevated miR-218 expression, and n3-PUFA supplementation reduced it [107].

Choi et al. investigated the effects of n3-PUFA on postpartum depression in dams from the same experimental design [108]. The dams received either control or supplemented diets from gestation onward. Dams were separated from their pups for 3 h per day from PND 2–14. Post-weaning, they were subjected to behavioral tests assessing depressive behavior. Similar to the previous report, stress increased hippocampal expression of miR-218 while n3-PUFA supplementation mitigated the stress effects [108]. These two reports implicate miR-218 involvement in serotonergic signaling and HPA axis dysregulation associated with depressive phenotypes [107,108].

**Figure 5:**
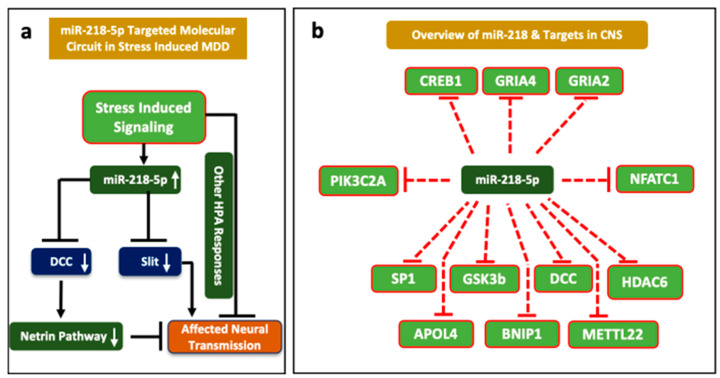
miR-218-5p targeted molecular circuit in stress-induced neuropsychiatric conditions. Stress-induced upregulation of miR-218 causes deficiency in Netrin and Robo signaling pathways via DCC and SLIT genes (**a**). The enriched targets of miR-218 in the central nervous system (**b**).

Rocchi et al. reported that miR-218 is involved in glutamatergic signaling. Primary hippocampal neurons of C57B16/J mice were cultured. Their expression of miR-218 was modulated by transfection with vectors for miR-218 expression or inhibition. Transduction with a lentiviral vector for miR-218 overexpression or miR-218 sponge was also employed to validate the biological mechanism. It was found that miR-218 interacts with the 3′ UTR of GRIA2, a gene coding for the GluA2 subunit of AMPA receptors, and enhances its translation and expression [29]. Altogether, the above-described studies clearly show that miR-218 is regulated by stress and is involved in stress-related disorders such as depression (Figure 5a,b).

## 7. Conclusions

Empirical studies conducted so far illustrate a formidable role for miR-218 in the central nervous system. miR-218 has been associated with essential signaling pathways such as glucocorticoid signaling [32,33], serotonergic signaling [107,108], and glutamatergic signaling [29]. Thus, miR-218 regulation impacts stress response, HPA axis function, and related brain functions. The Netrin-1/DCC signaling pathway, along with the others, impacts neuronal development and synaptic plasticity [31]. MiR-218 is expressed throughout the brain, notably in the hippocampus and PFC [30]. It is expressed at various levels through life stages as seen by adolescent and adult animal models, which again implicates its role in development [89]. With these findings considered, it is becoming increasingly clear that miR-218 plays a role in the pathophysiology of neuropsychiatric disorders, notably stress-related disorders such as MDD [33]. One detail that needs to be addressed is how some studies may have seemingly contradictory findings. For example, Dwivedi et al. found upregulated miR-218 expression in the mPFC of CORT-treated rats [32]. Meanwhile, Torres-Berrio et al. found downregulated miR-218 expression in the mPFC of CSDS mice [28]. However, different models were used, which may explain the differences. Corticosterone injection is used to artificially activate glucocorticoid signaling, resulting in stress response, while CSDS allows for a more general stress response [109]. Additionally, these studies focused on different signaling pathways, GR signaling or Netrin-1/DCC signaling [110,111]. It is important to consider that the participation of miR-218 in various signaling pathways may have varied outcomes [112,113]. It is imperative to evaluate the causality of these interactions whether miR-218 dysregulation contributes to a dysregulated stress response or is a compensatory mechanism to an abnormal stress response.

It would also be interesting to thoroughly evaluate how the effects of miR-218 expression change throughout an individual’s development. Since miR-218 expression affects neuronal development and plasticity, it is expected that miR-218 expression levels will change over the course of development. This concept has been broached using adolescent animal models. Torres-Berrio et al. reported an increase in miR-218 expression from adolescence to adulthood. While their studies found that increased miR-218 expression in adulthood would bolster stress resilience, they found that higher than usual, or “adult-like” levels in adolescence, would negatively impact development and make the animal stress-susceptible in adulthood [89]. The reports from Kim et al. and Choi et al. also demonstrate that modulation of miR-218 expression at various stages of life can impact the development of depressive phenotypes [107,108]. Alongside developmental-stage differences, it would be interesting to know whether dysregulation of miR-218 occurs parallelly or perhaps conversely in different brain regions [114]. MiRNAs may have specialized effects based on the brain region in which they are expressed. While studies have examined either the PFC or the hippocampus, it may be useful to have studies that examine changes in miRNA expression in both brain areas at the same time in the same animal model [115].

## 8. Future Directions

Currently, there are a limited number of studies reporting on the role of miR-218 in depression, and more research needs to be performed on the subject. Most studies thus far use animal models or cell culture [32,33,89,106]. Only a few studies have used human patient peripheral tissue or postmortem brain samples to validate the findings from animal models [28,31,106,116]. Hopefully, future studies can be performed with human patient samples to corroborate findings in animal models. Once the role of miR-218 in the pathophysiology of MDD is more thoroughly characterized, its potential as a therapeutic target for future treatments can be evaluated. While postmortem tissue is not readily available, particularly in adolescent studies, peripheral tissue has the potential to serve as a suitable proxy. For example, Torres-Berrio et al. correlated the expression of miR-218 circulating in the blood with mPFC expression using animal models [28]. This process can be taken a step further by isolating brain-derived exosomes from patient blood samples. These small, extracellular signaling vesicles released from neurons in the brain for communication can more accurately reflect the levels of expression present in the central nervous system [117]. In this way, we can explore the viability of miR-218 as a biomarker for depression. More studies are also needed to examine the other targets of miR-218. For example, this miRNA represses the NF-kB signaling pathway and TNF receptor [97,118]. On the other hand, miRNA-218 inhibits type I interferon production and facilitates virus immune evasion via targeting RIG-I [102]. As mentioned above, miR-218 overexpression increases glutamatergic synaptic transmission at both single-neuron and network levels and plays a key role in the regulation of AMPA-mediated excitatory transmission and the homeostatic regulation of synaptic plasticity [29]. These mechanisms could also contribute directly or indirectly to changing brain functions and behavior in various neuropsychiatric disorders.

So far, there is no study examining the sex-specific effects of miR-218 in psychiatric disorders. Since MDD and anxiety are more prevalent in females than males [119,120,121], future studies are needed in this direction.
